# Ethical examination of AI coaches: privacy, bias, and responsibility

**DOI:** 10.3389/fdgth.2026.1781352

**Published:** 2026-03-30

**Authors:** Wei Li, Liang Bian, Wei Wu

**Affiliations:** 1 School of Marxism Studies, Shanghai University of Sport, Shanghai, China; 2 School of Marxism, Shanghai University of Finance and Economics, Shanghai, China; 3 Academy of Marxism, Nanjing University of Finance and Economics, Nanjing, China

**Keywords:** AI coach, ethical risks, fairness, personalized training, sport

## Abstract

The integration of artificial intelligence (AI) into sports, particularly through AI-driven coaching systems, marks a transformative advancement with the potential to revolutionize personalized training. AI coaches can create customized, data-driven training programs designed to optimize athletic performance. However, this technological progress also brings with it significant ethical concerns, including privacy violations, data biases, and ambiguous responsibility in cases of failure or misuse. These risks extend beyond societal norms, posing threats to fundamental personal rights and raising questions about the fairness of athletic competitions. For example, privacy breaches could expose sensitive athlete data, while biases in training algorithms may create unfair advantages or disadvantages. Moreover, the lack of clear accountability for AI-related failures may lead to difficult legal and ethical dilemmas. To address these challenges, it is crucial to implement robust ethical safeguards. These safeguards should prioritize enhanced privacy protections, ensure equitable data collection and processing, and establish clear guidelines for the allocation of responsibility. By implementing such measures, AI coaches can be developed in a way that is ethically responsible and socially beneficial, thereby maximizing their potential to positively impact sports training.

## 1 Introduction

Since 2017, research in the field of AI in sports has experienced substantial growth (1). However, the ethical implications of AI in sports have not received sufficient attention. This area of research is critical because it not only addresses the boundaries and regulations governing the technology's application but also has a direct impact on the core values that underpin human engagement in sports activities, such as fair competition and privacy protection. Consequently, this field urgently requires further investigation and comprehensive analysis.

Sports training is a specialized process (2), where individuals, under the guidance of professional coaches, can reap numerous benefits, including maintaining physical fitness and enhancing immunity. However, the cost of hiring a professional coach is often prohibitively high for many, leading most individuals to rely on self-guided exercise routines. In response to this challenge, Artificial Intelligence Coaching (hereafter referred to as “AI Coaching”) has emerged as an innovative solution, integrating AI technology into sports training. AI Coaching provides personalized exercise advice and real-time guidance, helping individuals achieve optimal training outcomes.

Artificial intelligence (AI) has significantly transformed the sports landscape, redefining how athletes, coaches, referees, fans, and other stakeholders engage with the sport. For example, AI can analyze athlete data to create personalized training plans (3), assist coaches in making more informed decisions during competitions, and enable referees to “learn” and officiate in ways similar to humans by training algorithms on large datasets (4). Additionally, AI enhances fan engagement by providing immersive experiences through virtual reality (VR) and offers teams valuable insights into market trends, branding, and player transactions. Despite considerable investments in advancing AI technologies within the sports sector, there remains a significant gap in research addressing the ethical implications of these applications, particularly in the context of AI-driven coaching, which could lead to potentially serious negative consequences (5).

Nevertheless, as with any technology that has a profound societal impact, the ethical risks associated with AI in sports extend beyond the immediate context of the game itself, affecting all participants. The key ethical concerns related to AI coaching include privacy violations, data bias, and the uncertainty surrounding the attribution of responsibility. To address these challenges, this paper proposes strategies for promoting the responsible use of AI in sports, ensuring that individuals can enjoy both the benefits of technological advancement and the foundational values of sport.

This article is a conceptual and normative ethical study that integrates both aspects. By analyzing and synthesizing relevant literature on topics such as artificial intelligence, digital sports, AI coaches, and the intersection of AI and sports, it identifies the core technical features, application scenarios, and potential ethical risks associated with AI coaches. Additionally, the article uses the case of athletes employing AI coaches for personalized training as an illustrative example, ensuring that the argument is grounded in actual technological foundations.

This article begins by introducing AI coaches and demonstrating their technical foundation through a case study of an AI coach providing personalized training to athletes (Section 2). It then analyzes the three major ethical risks associated with AI coaches in sports: privacy violations, data bias, and unclear responsibility attribution (Section 3). In Section 4, the article proposes corresponding solutions, including privacy protection strategies, bias mitigation mechanisms, and responsibility allocation frameworks. Additionally, it emphasizes that the sustainable development of AI coaches requires coordinated participation and oversight from multiple stakeholders.

## 2 Artificial intelligence coach

In this article, an artificial intelligence coach is defined as a fully autonomous AI system equipped with both an adaptive learning system and a tracking system. A fully autonomous AI refers to an AI that can autonomously establish or modify its ultimate goals based on its cognitive development. Its autonomy is reflected not only in executing preset goals but also in its ability to independently determine and adjust these goals (6). The AI coach can interact with trainees, modify their cognition and behavior during exercises, provide customized training suggestions aligned with the trainees’ goals, create personalized exercise content, and continuously adjust the training plan based on the trainees’ actual progress.

To provide a detailed and specific explanation of the core function of an artificial intelligence coach, this article will present a case in which an AI coach conducts personalized training for athletes (7, 8). The selection of this case does not suggest that it encompasses all potential application scenarios for AI coaches, but rather that it effectively illustrates the technologies used by AI coaches, such as data perception, data fusion, decision-making, human-computer interaction, state recognition, and the dynamic optimization of training plans. These technologies serve as the common technical foundation shared by AI coaches, whether applied in rehabilitation, fitness, or competitive settings.

The AI coach supports athletes in their personalized training through the following six steps:

1. Planning: The AI coach analyzes the athlete's historical performance, physiological indicators, injury history, personal goals, and other relevant data to automatically generate a highly personalized training plan. The coach can conduct an online review and make adjustments to the plan, which are then synchronized with the athlete via the platform. The athlete receives and confirms the training plan on their mobile device, enabling a collaborative approach to goal-setting for the training regimen.
2. Initiation: At the beginning of the scheduled training session, if data from sensors (such as those in smart wearable devices) or application logs indicate that the athlete has not actively started the training, the AI coach will send a friendly reminder via the mobile device to confirm the athlete's training intentions and status, helping them overcome inertia.
3. Introduction: Before each training session, the AI coach provides a clear demonstration of the movement techniques and technical standards for the lesson using videos, 3D animations, or augmented reality (AR). Athletes do not need to memorize complex instructions; instead, they can intuitively grasp the specific content and proper techniques for each session, which helps reduce their cognitive load to some extent.
4. Monitoring: During training, the AI coach uses computer vision (e.g., the phone camera) and wearable sensors to monitor the quality of the athlete's movements in real time. This includes assessing the accuracy of movement trajectories, timing of exertion, joint angles, stability, and detecting any compensatory movements. If the coach detects that the athlete's movement quality falls short of expectations, the AI will immediately provide corrective feedback through screen prompts, voice instructions, or tactile cues (such as the vibration of a smart wristband). Additionally, when the athlete performs the movements correctly, the AI coach will offer positive reinforcement.
5. Adapting: The AI coach continuously monitors the athlete's real-time physiological data (such as heart rate), fatigue levels, and movement quality. If the AI coach detects signs of fatigue or abnormal movements, it will prompt the athlete about their training status in real-time through voice or interface interaction. The coach will then intelligently adjust the number of training sets, intensity, or recommend an increase in rest time as needed.
6. Conclusion: After the training session, the AI coach automatically generates a detailed report, which includes the training volume, technical quality score, physical exertion, and a comparison with historical training data. Based on this performance, the AI coach updates the athlete's ability model and adjusts the long-term training plan for future sessions. Finally, the AI coach confirms the next training schedule with both the athlete and the coach.

The primary role of human coaches is to help trainees achieve performance levels that would otherwise be unattainable. Coaches also have a significant impact on both the physical and psychological development of their trainees (9). The process outlined earlier, where artificial intelligence coaches facilitate personalized training for athletes, demonstrates that AI coaches can offer a customized experience similar to that of on-site coaches. By processing large volumes of patient data for realtime analysis and feedback, AI coaches not only motivate patients to engage with challenging tasks but also provide emotional support during setbacks. Furthermore, AI coaches continuously adapt to the trainee's specific needs, ensuring personalized guidance and support that evolves over time with the trainee's experience and progress (10).

Thure Georg Weimann et al. distinguish three types of coaching: face-to-face coaching, remote coaching, and coaching by autonomous systems (11). Face-to-face coaching involves real-time interaction and guidance with the trainee in person. This traditional method typically occurs in gyms, sports fields, or private training rooms, where the coach can make adjustments based on the trainee's performance. The advantages of face-to-face coaching include enhanced motivation, engagement, and real-time monitoring to ensure correct movements and reduce the risk of injury. Remote coaching occurs through video conferencing or recorded videos, allowing the coach and trainee to be in different locations. This method enables trainees to access coaching regardless of geographic limitations and is often more affordable than in-person sessions. It also provides the added benefit of reviewing past sessions for further learning. Autonomous system coaching relies on an app or web platform to guide the trainee through a pre-set program. This method is suitable for self-directed trainees, allowing them to train at their own pace. By recording and analyzing data, autonomous systems offer insights into the program and adjust it based on the trainee's needs. These three coaching methods can be combined or selected based on the trainee's preferences and requirements to maximize training outcomes.

As demonstrated in the case studies discussed earlier, artificial intelligence coaches primarily exhibit characteristics similar to those of human coaches. From a positive impact perspective, the AI coaches, as illustrated in these cases, offer precise, customized movement correction suggestions and emotional feedback aligned with the principles of positive psychology, all based on real-time performance data from athletes. Furthermore, as a consistently reliable and non-judgmental interactive entity, the AI coach provides a degree of procedural security and companionship (12). The AI coach's capabilities in personalized training planning, task execution, and emotional feedback mirror those of human coaches, indicating its potential to effectively train athletes.

In addition, artificial intelligence coaches are capable of delivering remote coaching and training through autonomous systems. Through apps, AI coaches can simulate aspects of remote coaching, such as teleconferencing, offering greater flexibility. AI coaches are highly accessible and flexible, both in terms of time and location, making them cost-effective in terms of both time and financial investment (13). Furthermore, AI coaches can provide personalized training plans, feedback, and data analysis similar to autonomous system coaching. As a result, AI coaches represent a hybrid model that combines the benefits of all three coaching types to offer a flexible and efficient training solution. Additionally, AI coaches continuously record, monitor, and assess the emotional and behavioral states of trainees, allowing for the provision of valuable training suggestions. Ultimately, AI coaches are seen as a tangible entity, with users expressing a sense of security during their interactions, as they perceive themselves to be engaging with a non-judgmental entity (12).

However, it is important to note that while artificial intelligence coaches can provide emotional support and feedback to athletes, there remains a distinction when compared to the emotional connection that human coaches establish with athletes over the course of long-term training. The emotional feedback provided by AI coaches is derived from data analysis and algorithmic simulations, and its primary objective is to enhance athletic performance, rather than to replicate the genuine emotional understanding and care that human coaches offer. Therefore, although AI coaches may exhibit certain similarities to human coaches, they cannot be considered equivalent to them. The focus should be on the potential for AI coaches to create a new model of human-machine collaboration in training. Fully recognizing the profound impact of AI coaches is a crucial step in ensuring that AI technology can effectively support athlete training.

## 3 Three ethical risks: invasion of privacy, data bias and unclear attribution of responsibility

The emergence of AI coaches has revolutionized training techniques in modern sports, but it also introduces a complex set of ethical risks. These include concerns over invasion of privacy, data bias, and unclear responsibility attribution—three of the most critical ethical issues when evaluating the impact of AI coaching. First, privacy invasion poses risks related to the misuse of sensitive data, violating individuals’ privacy rights. Second, data bias undermines the fairness and effectiveness of AI coaching, potentially exacerbating social inequalities. Lastly, ambiguity in responsibility attribution makes it difficult for victims to seek justice. Compared to other ethical concerns, such as over-reliance on technology or suboptimal user experience, these risks have far-reaching legal, social, and moral consequences, as they directly affect fundamental rights and the fairness of sports competitions. Therefore, it is crucial to address these ethical risks and ensure that AI coaches uphold human values and moral standards while driving technological innovation. Only through a comprehensive understanding of and response to these ethical concerns can we protect the interests of athletes and facilitate the responsible development of AI coaching technologies.

### 3.1 Invasion of privacy

One of the key advantages of artificial intelligence coaches is their ability to continuously collect and analyze personalized data from athletes, which enhances the scientific precision of training regimens. To create an effective training plan, AI coaches require access to extensive personal data, typically gathered through wearable devices or mobile applications. This data encompasses a wide range of information, including, but not limited to, personal identification details such as name, date of birth, ID number, and contact information; health metrics such as weight, height, heart rate, and medical history; location data, including frequently visited places and exercise routes; and behavioral patterns, such as eating habits, exercise routines, and sleep schedules. Furthermore, AI coaches continuously monitor and assess the physical and athletic performance of athletes, adjusting training plans based on data-driven insights and correlations.

Through this comprehensive approach, the artificial intelligence system can generate personalized recovery plans aimed at optimizing rehabilitation and maximizing the athletic potential of individuals. Additionally, the AI coach can proactively identify potential injury risks and recommend preventive measures. This anticipated strategy provides a scientific foundation for developing effective training programs, ultimately enhancing athletic performance. While the data collection capabilities of the AI coach offer numerous advantages, their implementation also introduces significant risks, particularly concerning privacy violations, which represent a critical ethical concern.

From the perspective of artificial intelligence ethics, the definition of privacy is continuously evolving with advancements in technology. In earlier studies, privacy was often understood as an individual's state of being free from observation or interference, or from public attention (14). In the information age, the concept of privacy has increasingly focused on information privacy, which refers to an individual's ability to control their own data (15). With the widespread adoption of artificial intelligence systems, the concept of privacy has evolved beyond the protection of static data to encompass the safeguarding of dynamic data as well. Building on the aforementioned perspective of privacy, artificial intelligence coaches face significant challenges at each stage of data collection, processing, and application. Specifically:

Vulnerabilities in the data storage system could be exploited by hackers, potentially resulting in the unauthorized leakage of sensitive information. The consequences of such a data breach are severe. First, there may be instances of unauthorized use of the data. Ideally, the AI coaches should use athletes’ data exclusively for legitimate purposes, such as enhancing training quality and improving performance. However, if AI coaches exploit athletes’ data without explicit consent—such as for advertising or commercial purposes—this constitutes a violation of privacy rights and could result in financial harm. Second, competitors could gain an unfair advantage. If sensitive training data are leaked or accessed illegally, competitors could analyze athletes’ training patterns, physiological conditions, and weaknesses, enabling them to devise targeted strategies that elevate their competitive edge and undermine the fairness of the competition. Third, athletes may experience increased psychological stress. Disclosure of health data or training weaknesses could erode self-confidence, diminish competitiveness, and ultimately impair their athletic experiences and career development. Finally, location data collected by AI coaches presents significant security risks. The leakage of this information could expose athletes to potential threats such as tracking, harassment, theft, or physical harm.

In conclusion, artificial intelligence coaches can significantly enhance athletes’ training activities; however, they also present complex and serious risks related to privacy infringement. Clearly, the risk of privacy violation is not merely a theoretical concern; rather, it is directly tied to athletes’ rights and interests, the fairness of competitions, and personal safety. Therefore, it is essential to integrate privacy protection as a fundamental ethical principle into the design of AI coaching systems from the outset and to manage it through measures such as stringent laws and transparent data governance.

### 3.2 Data bias

Artificial intelligence coaches are also vulnerable to data bias, which is a critical ethical issue that significantly affects the effectiveness and fairness of training. As defined by Ntoutsi et al. (16), data bias in AI systems refers to the tendency or prejudice the system develops toward specific individuals or groups, which is considered unfair due to social and technical factors during the processes of data generation, collection, or processing. Consequently, data bias in AI coaching refers to inaccuracies or inequalities arising from the use of datasets that are insufficiently comprehensive or diverse, leading to disadvantaged groups. Specific manifestations of data bias include the following:

First, biases can result from imbalanced data collection methods. If AI coaches use datasets predominantly representing athletes from specific geographical areas, their training programs may disproportionately benefit individuals from those regions. As a result, when applied to athletes from different regions, the programs might not address their unique needs, reducing the overall effectiveness. For example, consider an AI coach that trains athletes based on data collected from individuals in Europe and the United States. The AI coach's recommendations regarding running posture, strength training, and other factors may be tailored to the average height, leg length ratio, and muscle mass typical of this group. However, this data may not be optimal for training plans designed for Asian athletes, who may have different average physical characteristics and strength levels. In other words, if Asian athletes follow the training data and plans intended for European and American athletes, it could increase the risk of physical injury, failing to address the unique physical conditions and needs of Asian athletes.
Second, biases in recommendation systems can arise. If AI coaches base training suggestions primarily on popular trends or data from highly active users, the resulting recommendations may not suit certain athletes, neglecting more appropriate training alternatives. For instance, while swimming—an energy-intensive, full-body workout—may be effective for weight loss, recommending it to a trainee who cannot swim would be inappropriate. The AI coach should suggest alternative aerobic exercises like running, cycling, or skipping rope instead.
Third, biases resulting from historical data imbalances may emerge due to the overrepresentation of specific demographic groups. For example, if an AI coach predominantly trains male athletes, the resulting programs will likely reflect male-specific needs and characteristics. As such, female athletes may receive programs that are not tailored to their unique requirements.

Just as privacy breaches can have severe consequences, data bias also leads to significant negative impacts. Initially, data bias compromises training outcomes. Effective programs need to be personalized to match each athlete's unique attributes, but a biased AI coach is unable to deliver properly targeted programs, reducing effectiveness and increasing the risk of injuries. Additionally, data bias may create an unjust distribution of resources. AI coaches may focus disproportionately on athletes who resemble the idealized profiles in the dataset, neglecting others who fall outside those parameters, thus fostering an inequitable training environment.

Furthermore, data bias can harm athletes’ psychological well-being. Athletes naturally seek fair treatment and personalized attention from their AI coaches. However, perceived biases in the allocation of resources or attention can negatively impact self-esteem, diminishing mental health and competitive performance. Finally, data bias erodes trust. Athletes who identify biases in AI coaching practices may perceive these systems as unfair, weakening their confidence in the technology and damaging the relationships between athletes and coaches (17). Ultimately, diminished trust in AI coaching impedes its broader adoption and effectiveness.

### 3.3 Unclear attribution of responsibility

The issue of responsibility attribution for artificial intelligence coaches arises from a fundamental theoretical debate: the definition of the responsible party's qualifications. Mark Coeckelbergh argues that artificial intelligence lacks true self-awareness and the ability to make moral decisions based on ethical reasoning, and therefore, the AI system itself cannot bear moral responsibility (18). In contrast, Hutan Ashrafian contends that for future strong artificial intelligence systems that may possess consciousness and rationality, the framework for responsibility can be redefined to assign responsibilities within a specific range that aligns with their capabilities (19). Therefore, in the specific context of artificial intelligence coaches, there is also the challenge of unclear responsibility attribution.

The ambiguity surrounding unclear liability attribution complicates the traceability of the accountability system and the protection of stakeholders’ interests. As previously discussed, specific actions of AI coaches—such as creating inappropriate training plans—can negatively affect athletes, leading to injuries or poor competitive outcomes. Therefore, when adverse events occur due to the decisions of an AI coach, it is essential to establish who bears responsibility, whether it is the developer, the AI coach itself, or the athlete.

If responsibility is placed on the developer, the developer might argue that the product was designed correctly and that harm resulted from the athlete's failure to adhere to the AI coach's guidelines. Conversely, if responsibility is assigned to the AI coach, it may argue that it was following the developer's programmed instructions, pointing to potential errors in the design or issues with the athlete's adherence. However, if the AI coach operates autonomously and independently generates training plans that result in harm to the athlete, the coach itself should bear responsibility. Autonomy, defined as the ability to “do otherwise,” underpins moral accountability (20).

Alternatively, if responsibility is attributed to the athlete, the athlete might challenge both the developer and the AI coach, arguing that the system was flawed or improperly designed. If the athlete adhered strictly to the AI coach's guidance, the resulting injuries would logically be attributed to deficiencies in the AI's advice.

This issue of unclear responsibility is a matter of risk transfer among three key stakeholders: developers, AI coaches, and athletes, each of whom may attempt to assign blame to another. Developers may argue that misuse by the athlete caused harm, AI coaches may point to errors in the developer's programming or the athlete's non-compliance, and athletes may claim that flaws in the AI system or improper guidance led to the damage.

As with privacy breaches and data bias, unclear responsibility attribution can lead to significant consequences. Ambiguity regarding accountability can spark legal disputes, where stakeholders accuse each other of negligence, raising litigation risks. These disputes can lead to legal conflicts over compensation, resulting in financial harm for both developers and athletes. Moreover, failure to properly assign responsibility can damage the reputations of AI developers and sports organizations, leading to negative publicity. In the long term, persistent uncertainty regarding liability may undermine athletes’ trust in AI coaching, inhibiting innovation and limiting its potential in sports.

In conclusion, the ethical risks surrounding privacy violations, data bias, and unclear responsibility attribution in AI coaching highlight the need for robust ethical frameworks. While AI coaching offers substantial potential benefits, failing to address these risks may result in physical harm, economic repercussions, and broader social inequalities. The next section will explore strategies to mitigate these ethical risks, ensuring that AI coaching technologies develop in an ethically responsible manner.

## 4 Ethical solutions

### 4.1 Comprehensive ethical governance for AI coaching

AI presents a unique opportunity to accelerate the growth of the sports industry. By practically applying AI, it delivers intelligent and refined sports products and services to consumers, meeting their demands and significantly advancing industry development (21). However, the integration of AI in sports introduces substantial ethical challenges, affecting not only the game itself but also the participants involved (22). Specifically, the widespread adoption of AI in coaching, decision-making, training, and analytics raises critical issues such as privacy violations, biases in data-driven personalized advice, and the ambiguity surrounding responsibility for incorrect decisions or inappropriate recommendations.

To effectively manage these complex ethical risks, appropriate countermeasures must be systematically developed and implemented. These measures should enhance privacy protection mechanisms, ensure fairness in data handling, and clarify responsibility allocation, enabling performance improvements without compromising the fundamental rights and interests of trainees. This will foster the ethical and safe utilization of AI coaching technologies.

### 4.2 Privacy-preserving design and data protection

The ethical approach to privacy risks posed by AI coaching involves implementing comprehensive strategies, ranging from minimal data collection to stringent encryption practices. Ann Cavoukian's advocacy of the principle “proactive rather than reactive, preventive rather than remedial” highlights the necessity of integrating privacy considerations into technology design from the outset, thus preventing privacy issues before they occur (23). Therefore, designers must anticipate and prevent potential privacy violations associated with AI coaches before these risks arise.

During the design and development stages of AI coaching systems, privacy protection should be a fundamental principle, embedded throughout the system to minimize the need for reactive solutions after privacy violations occur.

The principle of data minimization should govern data collection practices. This principle dictates that AI coaching systems should collect only the minimum amount of data required to achieve their functional goals. For example, when creating personalized training programs, it is appropriate to collect relevant data such as the trainee's weight, height, strength, speed, agility, and endurance. However, irrelevant personal data, such as family members’ names or vehicle license plates, should be excluded. Targeted data collection like this significantly reduces the risk of personal or familial data being compromised or misused for unauthorized commercial purposes in the event of a data breach.

Robust encryption and anonymization processes must be implemented for all collected data. Encryption ensures that trainee data is securely encoded and can only be accessed through a specialized data parsing system unique to the AI coaching platform. This way, even if a data breach occurs, unauthorized third parties will be unable to decipher the trainees’ identities or misuse their data. Additionally, anonymization techniques prevent direct association between the data and specific individuals, further improving data security and reducing the risk of misuse.

Moreover, increased transparency must be prioritized within AI coaching systems. Users of the system often lack knowledge about which data is being collected, how it is processed, and who it is shared with Jud and Thalmann (24).Transparency means clearly informing trainees about how their data will be collected, used, stored, and managed before initiating any coaching relationship. Trainees should have the explicit right to informed consent and control over their data, including the ability to view the data collected by AI coaches and exercise their right to delete it as needed. Specifically, trainees not only have the right to informed consent, but this consent must be a prerequisite for handling their sensitive health data. This enhanced transparency not only fosters trust and autonomy but also promotes responsible and ethical engagement between AI coaches and trainees.

It is important to emphasize that for artificial intelligence coaches to effectively implement data and privacy protection, relevant measures must be enacted under the oversight of global data governance regulations. This means that the entire data processing process must actively adhere to the compliance requirements of multiple jurisdictions, including UNESCO's Recommendation on the Ethics of Artificial Intelligence, the EU's General Data Protection Regulation, and China's Personal Information Protection Law. Additionally, the design and implementation of data and privacy protection mechanisms must be dynamically aligned with the rapidly evolving global AI regulations.

### 4.3 Fairness assurance and data bias mitigation

In addressing the risk of data bias introduced by AI coaching, it is important to note that “data bias is an unavoidable product of historical data itself” (25). However, several ethical countermeasures can be implemented to ensure multi-level interventions—specifically targeting data sources, detection, and correction—to minimize these risks. Bias auditing tools, which typically rely on a combination of various methods, can be used to detect and analyze biases in artificial intelligence systems. These methods include fairness metrics, counterfactual analysis, sensitivity analysis, algorithmic transparency, and adversarial testing (26).Open-source toolkits, such as Aequitas (27) and Fairlearn (28) also support the evaluation of data fairness and help mitigate data bias.

Diversity in data collection is essential. AI coaches must ensure diversity in the acquisition of training data, whether sourced externally or directly from trainees. Specifically, AI systems should avoid biases that result from imbalanced representation, ensuring no particular social group is disproportionately represented or neglected. An open-source toolkit, AI Fairness 360, which encompasses a comprehensive dataset and model metrics, is employed to detect, report, and mitigate discrimination and bias in machine learning models across the entire AI development lifecycle (29). For instance, training data should reflect equitable representation across diverse social backgrounds and cultural contexts, thus preventing scenarios where male trainees are disproportionately included compared to female trainees. The benefit of promoting diverse data collection is that it reduces the inherent risk of data bias, enabling AI coaches to offer equitable and unbiased training programs that are tailored to diverse trainee profiles.

Mechanisms for data bias detection and correction are also vital. AI systems must routinely audit existing datasets throughout the training plan development process. By employing methodologies such as bias detection algorithms, AI systems can identify and correct biased data, preventing such biases from permeating automated systems (30). Once biases are detected, actions such as data cleansing or resampling should be promptly undertaken to maintain unbiased integrity. This proactive detection and correction not only mitigates immediate bias concerns but also prevents more widespread issues in the training of future trainees.

Establishing a continuous monitoring and feedback mechanism is essential. In addition to the initial stages of data collection, detection, and correction, comprehensive oversight and response feedback channels must be integrated into the AI coaching system. Ongoing bias assessments should be performed throughout the AI model's lifecycle, including during preprocessing, algorithm adjustments, and post-deployment monitoring. Real-time monitoring of biases, coupled with the development of feedback and update mechanisms—including clear implementation protocols—should be established to address bias-related incidents promptly and transparently (31). For instance, monitoring involves closely examining the behavior of AI trainers to detect discriminatory practices or differential treatment among various learner groups. Upon identifying such issues, alerts should be sent to system developers or operators to implement corrective measures, such as adjusting training plans or suspending problematic sessions. The establishment of continuous monitoring and feedback ensures sustained impartiality throughout AI-guided training processes, enabling rapid identification and resolution of emergent biases.

Regarding the ethical risks associated with responsibility attribution in AI coaching, several ethical measures can help mitigate these concerns by establishing clear responsibility and accountability frameworks. First, explicit role definitions and responsibility allocation are essential. By clearly defining the responsibilities of developers, AI systems, and trainees, each stakeholder can effectively manage their duties, avoiding ambiguity or shifting of responsibility (32). For example, it is essential to clearly define the responsibilities of developers, operators, sports organizations, coaches, and athletes in legal contracts and industry regulations. The following arrangements can be made: developers are responsible for ensuring the basic security and fairness of the algorithm; operators are responsible for ensuring compliance, providing risk warnings, and facilitating user communication regarding artificial intelligence in their operational environment; sports organizations are responsible for overseeing the internal process of introducing AI coaches; coaches are responsible for making final training decisions; and athletes are responsible for reasonably adhering to the training guidance. Additionally, a multi-level governance system should be established. This system should include a collaborative governance framework consisting of an internal ethics committee, industry standard bodies, independent third-party audits, and government regulatory agencies, all of which are responsible for developing ethical standards, conducting technical certification, and overseeing auditing and supervision.

Establishing a transparent accountability mechanism is also essential to ensure that, in the event of a training-related incident, the performance of each party's obligations can be traced. This requires that, when trainees are harmed during AI-guided training, the issue can be promptly identified and investigated. Effective accountability mechanisms involve tracing problems by reviewing data sources, examining AI system decision-making processes, and assessing trainee engagement to accurately determine the responsible parties. Transparent accountability ensures that responsibilities are traceable, providing strong evidence in accountability processes. For example, training plans developed by AI coaches must be approved by human coaches before they can be implemented. High-risk actions or fatigue warnings must undergo manual review. Any recommendations regarding athletes’ return to the field after rehabilitation or adjustments to their medication must be confirmed by team doctors or other relevant professionals. In summary, while it is important to allow AI coaches to assist in creating training plans for athletes, these systems should never override the judgment of professionals. If athletes sustain injuries due to issues that require manual review, the responsible personnel should be held accountable without delay.

Establishing compensation mechanisms is equally important. These measures would provide restitution to trainees harmed due to developer or AI system negligence. Practical steps include collaborating with insurance providers to offer injury or accident coverage to trainees before they engage in AI-mediated training activities. This compensation framework not only eases the financial burden on affected trainees but also creates a safety net that reinforces developer accountability and responsibility.

From a temporal perspective, moral responsibility not only involves the prevention of foreseeable risks but also encompasses the anticipated responsibility for potential future consequences (expected moral responsibility). Prospective ethical responsibility involves anticipatory accountability for potential future consequences. It requires stakeholders to carefully assess the potential impacts and outcomes of their actions and technological applications, taking proactive steps to avoid harmful effects (33). Therefore, the moral design and implementation of AI coaching technology must strictly adhere to privacy protection measures, proactive strategies to reduce bias, and a clear accountability framework. This framework should include well-defined responsibilities, human supervision, and technical interpretability. Strict data encryption and adherence to data minimization principles are key to protecting trainee privacy. At the same time, diverse data collection methods and robust monitoring and feedback mechanisms actively foster fairness and mitigate biases. By institutionalizing human supervision, ensuring the traceability and interpretability of decisions, and enabling rapid and accurate accountability when issues arise, the interests of athletes can be effectively protected.

Additionally, fostering open communication channels and actively encouraging trainee feedback and participation will significantly contribute to the continuous improvement of AI system functionality, safety, and adaptability across diverse training scenarios. By implementing these comprehensive ethical measures, AI coaches can not only provide personalized training support but also make meaningful contributions to broader social equity.

## 5 Conclusion

The ethical implementation and deployment of AI hold the potential to bring substantial benefits to the field of sports. Therefore, there is no fundamental reason to categorically oppose the use of AI in athletic contexts (34). AI serves as a supplementary tool for sports coaches rather than a replacement. Its core value lies in data-driven analysis and personalized empowerment. However, humanistic traits such as empathy, psychological motivation, and leadership are at the heart of coaching and cannot be replicated by AI (35). High-quality coaching resources are not equally accessible to everyone. As this technology continues to be widely adopted and its costs decrease, AI-driven coaching may offer effective training solutions, particularly for individuals who lack professional guidance. However, the introduction of AI-driven coaching also raises significant ethical concerns, especially regarding the personal rights of trainees and the potential impact on their physical health and career trajectories.

The Collingridge Dilemma suggests that controlling the negative impacts of emerging technologies is more feasible at an early stage; yet, it is precisely during this phase that we often lack the necessary understanding to guide their development in a beneficial direction (36). Therefore, there is an urgent need to proactively address the ethical risks associated with AI coaching. In confronting these challenges, technological advancements alone will not suffice. Comprehensive ethical reflection and the development of systematic coping strategies are crucial. In the long term, it is crucial to enhance the ethical awareness of society as a whole, particularly among young people, to cultivate future developers and managers who will approach innovation with a responsible perspective.

The sustainable integration of AI in sports depends on thoroughly recognizing and addressing these ethical issues, ensuring that AI functions as a supportive tool that enhances, rather than undermines, the integrity of sports and the rights and interests of athletes. It is essential that governments, sports organizations, technology developers, academic institutions, and other societal sectors work closely together to establish a scientifically sound and effective governance framework for AI, proactively mitigating potential risks (37). Only through coordinated engagement and regulation from multiple stakeholders can we create a fairer, more transparent, and accountable environment for sports technology. Thus, the implementation of AI in sports will require more than just technological advancement.

## Data Availability

The original contributions presented in the study are included in the article/Supplementary Material, further inquiries can be directed to the corresponding author.
